# A Disruptive Technology in the Making

**DOI:** 10.1016/j.jaccas.2024.102808

**Published:** 2024-12-04

**Authors:** Vinh Nguyen, Kirtivardhan Vashistha

**Affiliations:** a Department of Cardiovascular Medicine, Baylor College of Medicine, Temple Campus, Temple, Texas, USA; b Department of Cardiovascular Medicine, Mount Sinai Morningside-West, New York, New York, USA

**Keywords:** cardiac MRI, cardiomyopathy, lipid storage disease

**Figure:**
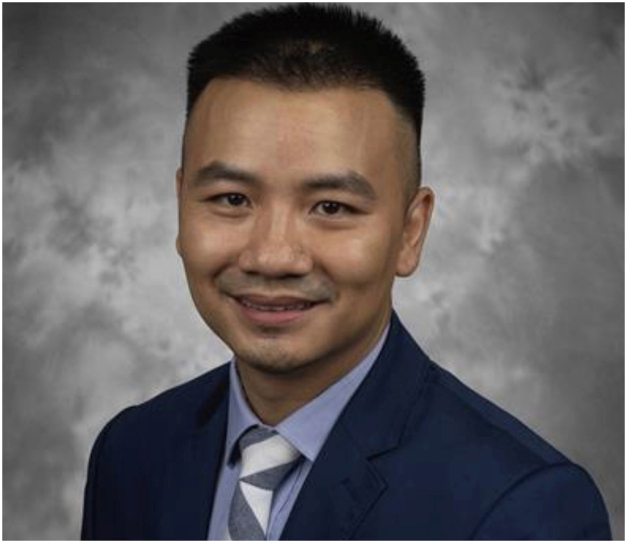

Imagine for a moment you’re living in the time of fur traders and trappers. You’ve spent years tinkering with the old, reliable “horse-drawn carriage” of cardiology: echocardiography. Sure, a buggy will get you there, but not with style. You eyeball wall thickness and M-mode, but you’re still riding on wooden wheels instead of the modern suspension. Imagine again that you’re transplanted to the era of the internal combustion engine. You went from a couple horses to hundreds and now have an unparalleled insight into the histological composition of the heart.

Cardiac magnetic resonance imaging has fundamentally transformed our understanding of myocardial structure. Gone are the days when measuring wall thickness is sufficient to declare hypertrophy. Current guidelines endorse the use of geometric formulas to calculate myocardial volume, which is then converted to mass by multiplying by the myocardial density (1.05 g/mL).^1^ However, when myocardial infiltration enters the scene, the validity of this formula is compromised because of alterations in tissue composition, and the myocardial density constant 1.05 g/mL no longer applies. An increased myocardial dimension should be termed "thickening" rather than hypertrophy, with an implicit footnote that there is more than meets the eye.

To appreciate how we got here, we must explore gadolinium-based contrast agents from an historical perspective. Initially introduced in the 1980s for tissue characterization, gadolinium-based contrast agents made areas of greater concentration appear brighter on T1-weighted sequences. By the 1990s, gadolinium-based contrast agents found their way into cardiac imaging in detecting myocardial infarction and fibrosis. In a landmark study, Kim et al^2^ described their use in myocardial infarction, showing that infarcted tissue retains contrast because of the ruptured cell membranes and expanded extracellular space, thus allowing for visualization of infarcted regions.^2^ By 2000, research had established that the degree of transmural late gadolinium enhancement (LGE) correlated with myocardial viability, cementing its role in the management of ischemic heart disease.^3^ Over the decade, LGE was expanded to myocarditis, dilated, hypertrophic, and infiltrative cardiomyopathies.^4^^,^^5^ Further development in the 2010s led to contemporary sequences such as T1 and T2 mapping and calculation of the interstitial and/or extracellular volume fraction.^6^ Although LGE marks myocyte loss and replacement fibrosis, elevated T1 mapping and extracellular volume fraction signify reactive fibrosis, which precedes replacement fibrosis and could still be a reversible.^7^

In this issue of *JACC: Case Reports*, Krogh et al^8^ present a case of a 38-year-old man with right arm weakness and paresthesia. Elevated serum creatine kinase hinted at a myopathic process, leading to a myocardial biopsy that revealed excessive lipid deposition. Genetic testing confirmed a diagnosis of neutral lipid storage disease with myopathy through identification of a missense mutation in the *PNPLA2* gene. Although T1 mapping was not available, it is important to note that this intracellular lipid vacuole accumulation *reduces T1 time*. Although systematic data on the natural history of neutral lipid storage disease with myopathy are lacking, it is plausible that its progression mirrors other fat-storage diseases, such as Anderson-Fabry disease. In these cases, T1 mapping is interpreted with caution. Initial lipid deposition reduces T1 time, which then rises because of an inflammatory response in the intermediate disease stage (*pseudonormalization of T1*). An advanced fibrotic stages is parallel by a *high T1 time*.

Several qualitative features are apparent on the LGE images in this case. First, the enhancement observed does not suggest complete replacement fibrosis given its intermediate signal intensity, which appears visually 4 to 6 SDs above normal myocardium. The degree of enhancement correlates with pathologic collagen deposition. Signal intensity can also be influenced by an increase in the cytoskeleton lattice, that is, pericellular, intercellular, and fascicular connective tissue.^9^ Complete replacement fibrosis, seen in myocardial infarction or advanced sarcoidosis, typically displays an intense LGE signal (>6 SD). Second, given the extensive cytoplasmic vacuole accumulation noted in histology, the hyperintense signal may also represent fat deposition, because fat similarly enhances on T1-weighted sequences. T1 mapping or fat saturation sequences could better differentiate whether this signal represents fat of fibrosis.

As we ponder the disruptive technology of cardiac magnetic resonance imaging, it is clear that its impact stems from its relatively “recent” emergence compared with echocardiography. Had the timelines been reversed, we might instead marvel at echocardiography’s efficiency in providing instant results through the Doppler effect. Regardless, there is no need for imagination to recognize the internal combustion engine as a transformative milestone in human evolution, just as magnetic resonance imaging is in the realm of cardiac imaging. So hop on the bandwagon; this disruption is only just the beginning.

## Funding Support and Author Disclosures

The authors have reported that they have no relationships relevant to the contents of this paper to disclose.
